# Mathematics Anxiety and Statistics Anxiety. Shared but Also Unshared Components and Antagonistic Contributions to Performance in Statistics

**DOI:** 10.3389/fpsyg.2017.01196

**Published:** 2017-07-24

**Authors:** Manuela Paechter, Daniel Macher, Khatuna Martskvishvili, Sigrid Wimmer, Ilona Papousek

**Affiliations:** ^1^Educational Psychology Unit, Department of Psychology, University of Graz Graz, Austria; ^2^Department of Psychology, Faculty of Psychology and Educational Sciences, Tbilisi State University Tbilisi, Georgia; ^3^Biological Psychology Unit, Department of Psychology, University of Graz Graz, Austria

**Keywords:** statistics anxiety, mathematics anxiety, gender, performance, effort motivation

## Abstract

In many social science majors, e.g., psychology, students report high levels of statistics anxiety. However, these majors are often chosen by students who are less prone to mathematics and who might have experienced difficulties and unpleasant feelings in their mathematics courses at school. The present study investigates whether statistics anxiety is a genuine form of anxiety that impairs students' achievements or whether learners mainly transfer previous experiences in mathematics and their anxiety in mathematics to statistics. The relationship between mathematics anxiety and statistics anxiety, their relationship to learning behaviors and to performance in a statistics examination were investigated in a sample of 225 undergraduate psychology students (164 women, 61 men). Data were recorded at three points in time: At the beginning of term students' mathematics anxiety, general proneness to anxiety, school grades, and demographic data were assessed; 2 weeks before the end of term, they completed questionnaires on statistics anxiety and their learning behaviors. At the end of term, examination scores were recorded. Mathematics anxiety and statistics anxiety correlated highly but the comparison of different structural equation models showed that they had genuine and even antagonistic contributions to learning behaviors and performance in the examination. Surprisingly, mathematics anxiety was positively related to performance. It might be that students realized over the course of their first term that knowledge and skills in higher secondary education mathematics are not sufficient to be successful in statistics. Part of mathematics anxiety may then have strengthened positive extrinsic effort motivation by the intention to avoid failure and may have led to higher effort for the exam preparation. However, via statistics anxiety mathematics anxiety also had a negative contribution to performance. Statistics anxiety led to higher procrastination in the structural equation model and, therefore, contributed indirectly and negatively to performance. Furthermore, it had a direct negative impact on performance (probably via increased tension and worry in the exam). The results of the study speak for shared but also unique components of statistics anxiety and mathematics anxiety. They are also important for instruction and give recommendations to learners as well as to instructors.

## Introduction

Statistics anxiety is a pervasive problem in the context of university studies, especially in social science degrees, such as psychology, education, or sociology (Onwuegbuzie and Wilson, 2003; Onwuegbuzie, 2004; Ruggeri et al., 2008). However, is it really statistics anxiety as a genuine form of anxiety that impairs students' achievements? Or is it rather that learners transfer previous experiences in mathematics and their anxiety in mathematics to statistics? The following study takes up this question and investigates the relationship between statistics anxiety and mathematics anxiety in a sample of psychology students in their first term.

### Statistics anxiety

Statistics anxiety describes the apprehension that an individual experiences in instructional situations, in evaluative contexts related to statistics, or when working on statistical tasks. It is an enduring, habitual type of anxiety (Onwuegbuzie and Daley, 1999; Onwuegbuzie, 2004). Consequently, students report manifold problems over the course of their statistics education (Macher et al., 2012).

Statistics anxiety influences performance in an examination but also during the preparation phase. In the examination, statistics anxiety is related to interference of task-relevant with task-irrelevant thoughts (Eysenck et al., 2007), such as worry and rumination, and reduces cognitive resources that are necessary for task solving. In a study by Macher et al. (2013), students assessed their statistics anxiety 2 weeks prior to the examination as well as their feelings of state anxiety at two points in time during the examination. Statistics anxiety instilled a high level of state anxiety at the beginning of the examination and was to a large degree responsible for the maintenance of a high anxiety level, which impaired academic performance.

These results suggest a negative influence of statistics anxiety. In contrast to this assumption, various studies found no or only low, insignificant correlations between statistics anxiety and academic performance. In their synopsis of 11 studies, Macher et al. (2015) explain these results with antagonistic, direct, and indirect effects of statistics anxiety. While direct effects of statistics anxiety in the examination are mostly harmful, indirect effects can be positive as well as negative for learning and performance. Negative effects prior to the examination mostly concern difficulties in time-management and procrastination during the preparation phase (Onwuegbuzie, 2004; Rodarte-Luna and Sherry, 2008; Macher et al., 2012). Procrastination was also negatively related to the use of deep-level cognitive strategies and meta-cognitive strategies (Wolters, 2003; Howell and Watson, 2007). However, statistics anxiety is also related to positive effects, such as increased effort as long as the anxiety level is not too high (Pekrun, 1988; Birenbaum and Eylath, 1994; Macher et al., 2015). Thus, negative and positive effects of statistics anxiety may outweigh each other.

Research studies suggest different antecedents that influence the development of statistics anxiety: Trait anxiety as a dispositional antecedent (Macher et al., 2013) can be described as the habitual tendency to perceive stressful situations as threatening (Spielberger, 1985; Endler and Kocovski, 2001; Meijer, 2001) and thus should be related to higher levels of statistics anxiety. Other studies found an influence of personal characteristics, such as gender, ethnicity, or age (Onwuegbuzie et al., 1997). In various studies, female students reported higher levels of statistics anxiety (Rodarte-Luna and Sherry, 2008; Macher et al., 2012, 2013, 2015; Papousek et al., 2012). Furthermore, situation-related antecedents, i.e., experiences and attitudes that result from statistics courses or courses in related knowledge domains, such as mathematics (Baloglu, 2003), are assumed to be related to statistics anxiety. In that sense, mathematics anxiety should also influence the development of statistics anxiety.

### Mathematics anxiety

Mathematics anxiety is regarded to be a widespread problem in school as well as in tertiary education. For example, Ashcraft and Moore (2009) estimate that 17% of the US-American population suffer from high levels of mathematics anxiety; studies in other countries arrive at similar estimations (Richardson and Suinn, 1972; Johnston-Wilder et al., 2014; Dowker et al., 2016).

Similarly to statistics anxiety, mathematics anxiety has been defined as feelings of apprehension and increased physiological reactivity when individuals have to manipulate numbers, solve mathematical problems, or when they are exposed to an evaluative situation which deals with mathematics (Hopko et al., 2003; Carey et al., 2016). Most studies and measurement instruments assume at least two (assessment-related) facets of mathematics anxiety: anxiety that is experienced when taking a test and anxiety experienced in classroom situations (Hopko et al., 2003; Cipora et al., 2015). Other studies add a content-related facet: numerical anxiety, which describes anxiety due to performing basic mathematics operations and manipulating numbers (Kazelskis, 1998; Baloglu and Zelhart, 2007).

Studies that investigated the effects of mathematics anxiety by standardized basic mathematics tests found task- and memory-specific effects. Individuals suffered a compromised working memory specifically in tasks, which need storing, updating intermediate results, and performing calculations and were less fluent in processing numbers (Cates and Rhymer, 2003; Ashcraft and Krause, 2007). Cipora et al. (2015) assume that feelings of stress and tension induce individuals to terminate the anxiety-evoking situation and thus tend to sacrifice accuracy for speed.

Mathematics anxiety does not only have direct effects on task performance but also influences learning and academic progress in a long range. Mathematics-anxious students tend to avoid mathematics-related situations and courses and more often exhibit procrastination behavior (Akinsola et al., 2007). In a study by Meece et al. (1990) the combination of a low academic self-concept in mathematics, low importance ascribed to mathematics, and mathematics anxiety influenced whether students in secondary education intended to enroll in mathematics courses or not. To our knowledge, only one study investigated the relationship between mathematics anxiety and performance in a statistics examination. Pletzer et al. (2010) found no correlation between both variables in the overall sample, but a significant correlation between mathematics anxiety and performance was found for a subgroup of students who responded to acute stress by increases in cortisol levels and subsequent decreases as the stressor (the examination) ended.

Several antecedents of mathematics anxiety have been identified. Gender is related to mathematics anxiety with females reporting higher levels of anxiety than males (Bieg et al., 2015; Cipora et al., 2015; Erturan and Jansen, 2015; Dowker et al., 2016). Yet, it is still debated whether females score higher than males on all facets of mathematics anxiety or whether differences can be mainly attributed to the test and course anxiety facets (Baloglu and Kocak, 2006). There is also mixed evidence concerning gender differences in performance. While some studies report differences in favor of males (Else-Quest et al., 2010; Cipora et al., 2015), others find no gender differences (Hembree, 1990; Devine et al., 2012; Erturan and Jansen, 2015). Altogether, it is advisable to control for gender differences in research studies on mathematics performance. Females do not only experience higher levels of mathematics anxiety but also show less self-confidence in their abilities in mathematics (Meece et al., 1990; Ertl et al., 2014). In addition, social determinants, such as the influence of parents, teachers, and peers as well as cultural stereotypes about giftedness in mathematics influence the degree of mathematics anxiety (Casad et al., 2015).

### Statistics anxiety and mathematics anxiety

Early research discussed whether statistics anxiety is really a construct separate from mathematics anxiety. It was argued that the contents underlying both types of anxiety form one domain of knowledge and cannot be regarded separately (Demaria-Mitton, 1987). Therefore, when initially introduced, statistics anxiety was treated as an affiliate function of mathematics anxiety. Recent studies consider statistics anxiety as being conceptually different from mathematics anxiety (Cruise et al., 1985; Baloglu, 2003). Of course, statistics uses basic mathematical concepts and calculations but its learning contents differ from mathematics in various aspects (Aksentijevic, 2015). Statistic tasks in majors, such as education, psychology, or sociology are more closely related to verbal reasoning (Buck, 1987), they require probabilistic reasoning processes, such as making inferences or drawing conclusions from data (Baloglu, 1999, 2003), and are often embedded into an applied context. Also, statistics anxiety is a widespread phenomenon among psychology novices across universities and nationalities, even though introductory statistics courses can greatly differ in how much mathematics they include (Ruggeri et al., 2008). These assumptions are supported by cognitive theories as well as brain functional studies on probabilistic and analytical reasoning. Evans (2003) and other authors (e.g., Goel and Dolan, 2003; Oaksford, 2015) distinguish two cognitive systems underlying reasoning: an evolutionary old system and an evolutionary recent and distinctively human system. Whereas, the former system is important for probabilistic reasoning, drawing heuristics, and intuitive understanding, the latter is associated with abstract reasoning and hypothetical thinking (Evans, 2003). Brain functional studies demonstrated evidence for this assumption, e.g., by using fMRI methodology (Goel and Dolan, 2003; Oaksford, 2015).

Yet, there is evidence that mathematics anxiety and statistics anxiety share at least some variance. In a study by Maysick (1984), statistics anxiety was a significant predictor of mathematics anxiety in multiple regression analysis (even though one might criticize that statistics anxiety was used as predictor and not as criterion variable). Zeidner (1991) reports a correlation of *r* = 0.41 between mathematics and statistics anxiety; however, only statistics anxiety and not mathematics anxiety correlated with grades in statistics. Birenbaum and Eylath (1994) report a correlation of *r* = 0.33, mathematics grades correlated significantly with both types of anxiety. Mathematics anxiety and statistics anxiety also correlated significantly and negatively with numerical ability, but only statistics anxiety correlated significantly with inductive reasoning ability. Solving tasks in statistics needs both abilities and draws on skills in the two types of cognitive processes described before.

However, studies on mathematics and statistics anxiety nearly exclusively used cross-sectional designs; many of them calculated mainly bivariate correlations between the two forms of anxiety but did not consider them within a context with mediating or moderating variables. Therefore, it is not yet clear whether statistics anxiety is only an after-effect of mathematics anxiety. Statistics anxiety might replace mathematics anxiety when a student no longer has to take courses in mathematics but encounters statistics tasks that seem to look like mathematics tasks.

### Present investigation: research questions and research models

The present investigation looks closer into the role of statistics anxiety and mathematics anxiety for learning and academic performance in statistics and investigates to which degree both concepts overlap or are different from each other. It will be surveyed how statistics anxiety and mathematics anxiety are related to specific learning behaviors and performance. Furthermore, antecedents of statistics anxiety and mathematics anxiety and their influence on learning and performance will be investigated. The following research questions will be focused on:

- Are statistics anxiety and mathematics anxiety independent concepts and/or do they overlap?
- How are antecedents of statistics anxiety and mathematics anxiety related to both types of anxiety?
- How are statistics anxiety and mathematics anxiety related to learning behaviors, such as procrastination and to performance in statistics courses?

To investigate the above research questions, three working models were developed and investigated by means of structural equation modeling. The variations of the model mainly reflect assumptions on the closeness of the relationship between mathematics anxiety and statistics anxiety.

**Figure 1:**
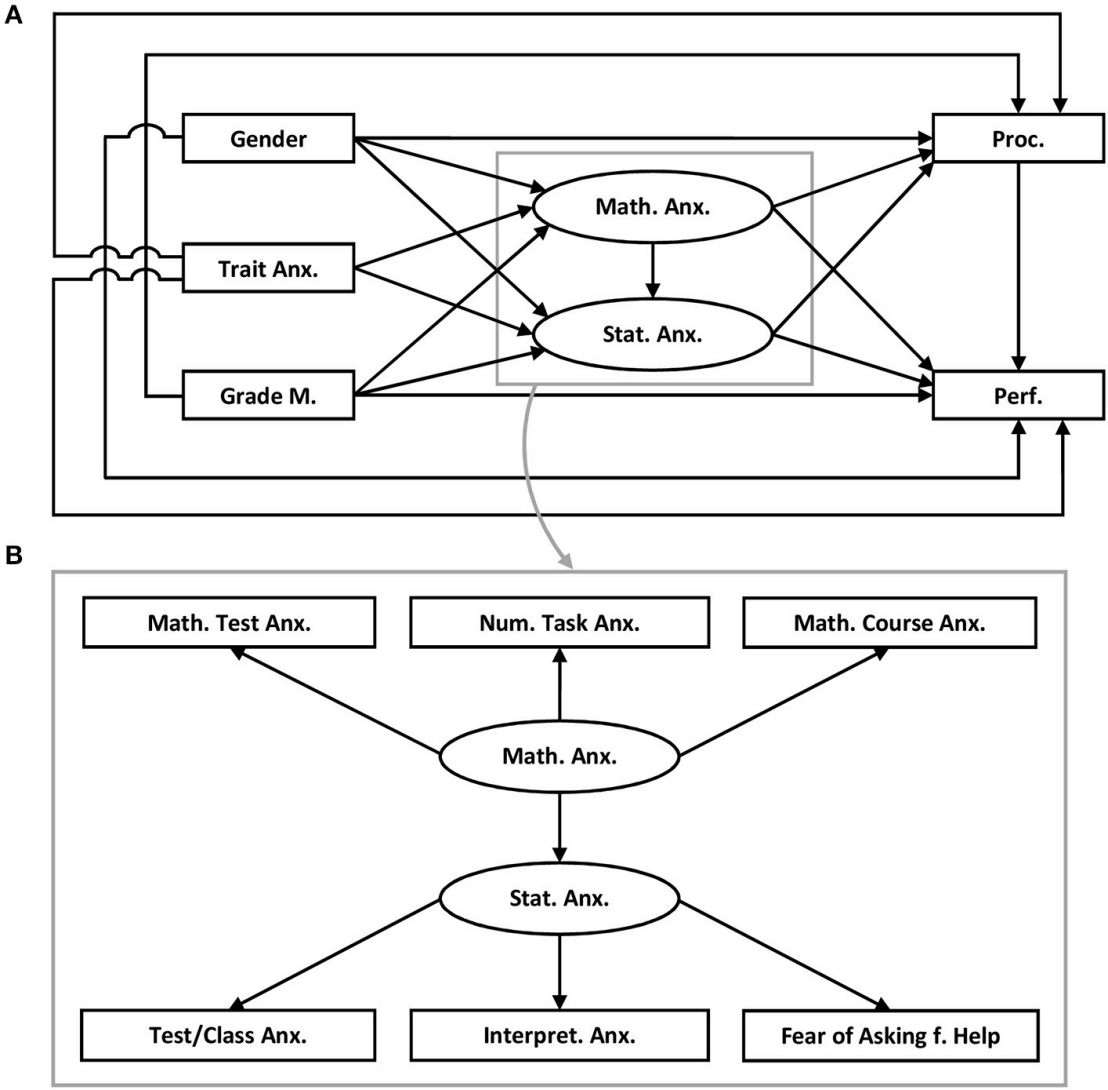
Model 1 **(A)** Trait Anx., Trait anxiety; Grade M., Grades in mathematics; Math. Anx., Mathematics anxiety; Stat. Anx., Statistics anxiety; Proc., Procrastination; Perf., Performance in the statistics exam; **(B)** Math. Test Anx., Mathematics test anxiety; Num. Task Anx., Numerical task anxiety; Math. Course Anx., Mathematics course anxiety; Test/Class Anx., Test and class anxiety; Intepret. Anx., Intepretion anxiety; Fear of Asking f. Help, Fear of asking for help.

Model 1 (compare Figure 1): Direct and indirect relationships between statistics anxiety, mathematics anxiety, learning behaviors, performance in the statistics examination, as well as direct and indirect relationships between putative antecedents of statistics anxiety and mathematics anxiety (gender, grades in mathematics at school, and trait anxiety) are investigated in the model.

Based on the studies described in the introduction, Model 1 assumes a relationship between gender, trait anxiety, grades in mathematics at school, mathematics anxiety, and statistics anxiety. Studies in which female students reported higher levels of domain specific anxiety than male students support this assumption (Rodarte-Luna and Sherry, 2008; Macher et al., 2012, 2013) as well as studies in which grades were negatively related to domain-specific anxiety (Baloglu and Kocak, 2006; Rodarte-Luna and Sherry, 2008; Macher et al., 2012, 2013). Trait anxiety, math anxiety, and knowledge in mathematics are regarded as antecedents of statistics anxiety. Based on findings in previous studies with samples at the same university but in different academic years, no differences between women and men in trait anxiety are assumed (Macher et al., 2012, 2013; Papousek et al., 2012).

Research has identified various variables that may influence performance in statistics as well as in mathematics. Generally, prior knowledge (e.g., measured by grades) is a strong predictor for later achievement (e.g., Paechter et al., 2015). Especially in domains like mathematics, science, etc. gender differences in achievement as well as in learning behaviors should be taken into account (e.g., Walsh and Ugumba-Agwunobi, 2002; Else-Quest et al., 2010; Macher et al., 2012, 2013). Learning behaviors, especially procrastination, are also influenced by traits and attitudes, such as anxiety (Onwuegbuzie, 2004; Rodarte-Luna and Sherry, 2008; Macher et al., 2012, 2013).

Research on statistics anxiety and performance suggests that indirect effects should be taken into account. In a synopsis of 11 studies by Macher et al. (2015), the majority of studies showed insignificant or very low bivariate correlations between these variables (e.g., Birenbaum and Eylath, 1994; Fitzgerald et al., 1996; Nasser, 2004; Lacasse and Chiocchio, 2005; Chiesi and Primi, 2010; Macher et al., 2012, 2013) but significant suppression effects between statistics anxiety, performance, and other variables.

A crucial part of Model 1 concerns the relationship between mathematics anxiety and statistics anxiety (see Figure 1B; Figure 1B is part of the model described in Figure 1A; for the sake of clarity, the structure of the latent variables with their facets were depicted in a separate panel). Model 1 regards mathematics anxiety as an antecedent and, therefore, as a predictor of statistics anxiety (Baloglu and Kocak, 2006). Both forms of anxiety are regarded as two separate concepts that, however, may have shared components. This assumption can be explained by the importance of different cognitive abilities in the two knowledge domains (e.g., inductive reasoning ability: Birenbaum and Eylath, 1994; Goel and Dolan, 2003; probabilistic and analytical reasoning: Evans, 2003; Oaksford, 2015). In the model, mathematics anxiety and statistics anxiety each form a separate latent factor with different facets. Each factor may be related to learning behavior and performance.

**Figure 2:**
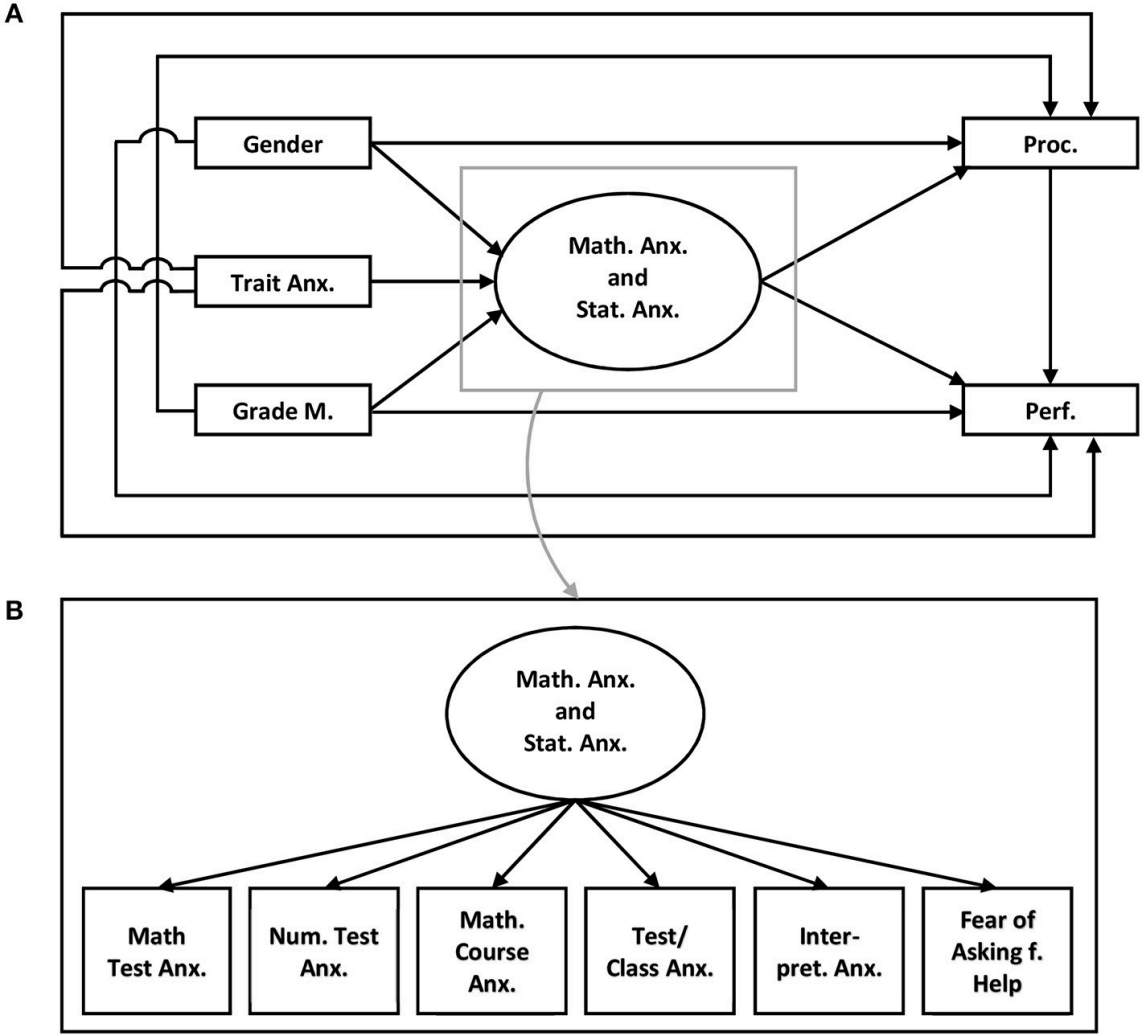
Model 2 **(A)** Trait Anx., Trait anxiety; Grade M., Grades in mathematics; Math. Anx. and Stat. Anx., Mathematics anxiety and Statistics anxiety; Proc., Procrastination; Perf., Performance in the statistics exam; **(B)** Math. Test Anx., Mathematics test anxiety; Num. Task Anx., Numerical task anxiety; Math. Course Anx., Mathematics course anxiety; Test/Class Anx., Test and class anxiety; Intepret. Anx., Intepretion anxiety; Fear of Asking f. Help, Fear of asking for help.

Model 2 (compare Figure 2): Model 2 differs from Model 1 only with regard to the assumed relationships between mathematics anxiety and statistics anxiety. It assumes that statistics anxiety is an affiliate function of mathematics anxiety (Demaria-Mitton, 1987). The different facets of mathematics and statistics anxiety are subsumed under one latent factor that may be related to learning behavior and performance.

**Figure 3:**
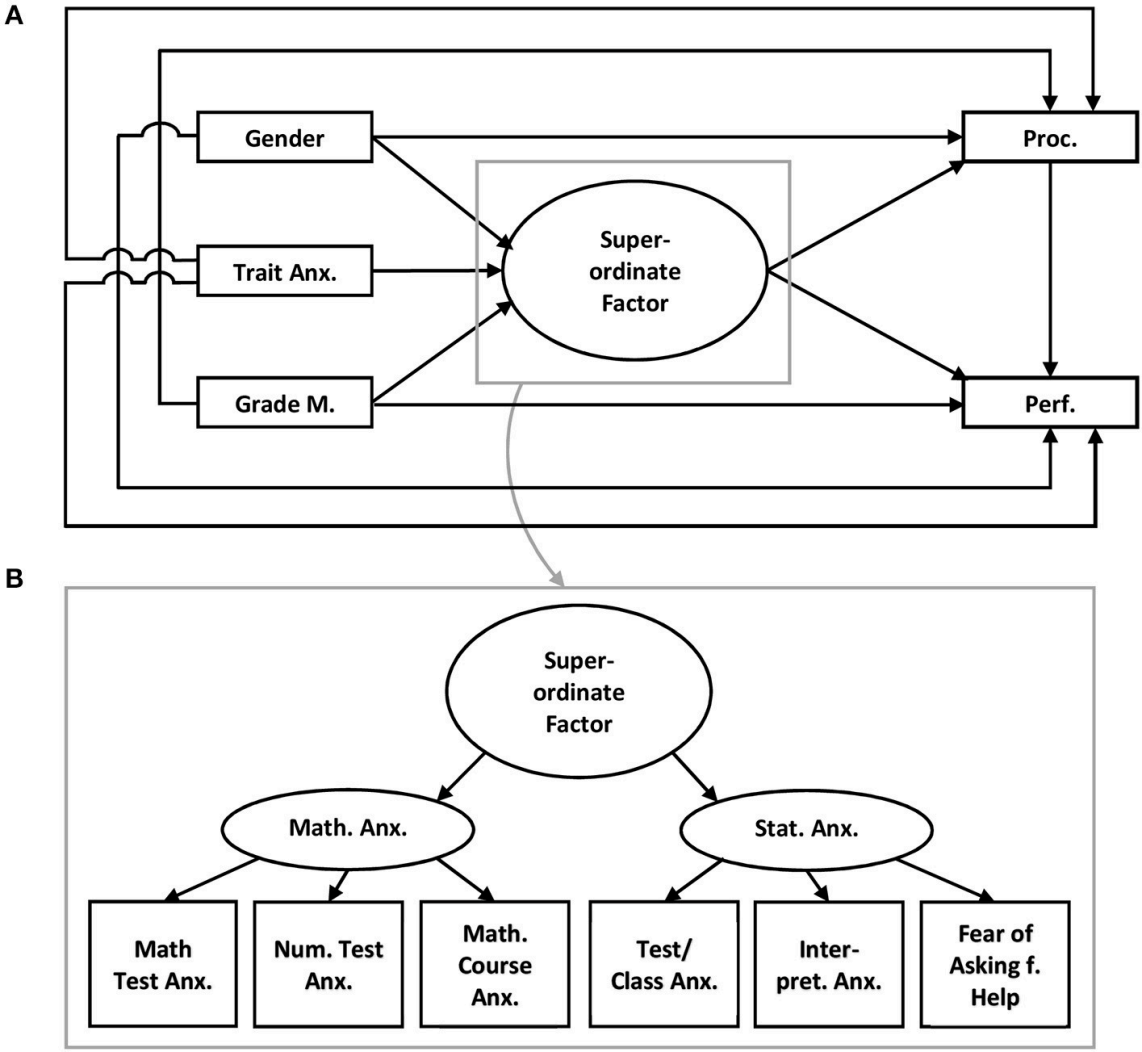
Model 3 **(A)** Trait Anx., Trait anxiety; Grade M., Grades in mathematics; Proc., Procrastination; Perf., Performance in the statistics exam; **(B)** Math. Test Anx., Mathematics test anxiety, Num. Task Anx., Numerical task anxiety; Math. Course Anx., Mathematics course anxiety; Test/Class Anx., Test and class anxiety; Intepret. Anx., Intepretion anxiety; Fear of Asking f. Help, Fear of asking for help.

Model 3 (compare Figure 3): Model 3 assumes that mathematics anxiety as well as statistics anxiety are separate latent factors with different facets and both latent factors belong to one superordinate factor which may be related to learning behaviors and performance.

## Method

### Participants

Participants were 225 undergraduate psychology students at the University of Graz, Austria; 164 students were female (72.88%) and 61 were male (37.22%). The gender composition of the sample corresponds to the gender distribution of undergraduate psychology students in Austria. The age of the participants ranged from 18 to 39 years (*M* = 20.44, *SD* = 2.89). The majority of students (95%) were between 18 and 25.75 years old. All participants were enrolled in an introductory lecture-based statistics course offered for psychology undergraduates in their first term. Course contents included topics, such as scales of measurement, different kinds of descriptive statistics and their interpretation, the fundamental principles of inferential statistics, basic univariate tests, and interpretation of results. The lecture lasted one term (approximately 15 weeks) and ended with a written examination in the last week of the course. Passing this statistics examination is a requirement for enrolment in the second part of the course as well as for various other subsequent courses in the study of psychology. All participants took part in the written examination. The study was performed in accordance with the 1964 Declaration of Helsinki and the American Psychological Association's Ethics Code. Review and approval was not required for this study in accordance with the national and institutional requirements. Participation in the research was voluntary. All participants gave written consent to participate in the study and to confirm that their data were used in an empirical study.

### Measures

Statistics anxiety was measured by the German version of the Statistics Anxiety Rating Scale (STARS; Cruise et al., 1985; Papousek et al., 2012). Three subscales of the STARS measuring anxiety were used; test and class anxiety with 8, interpretation anxiety with 11, and fear of asking for help with 4 items. The self-report items require participants to describe on a five-point scale ranging from “no anxiety” (1) to “very much anxiety” (5) how they feel in specific situations, such as “walking into the room to take a statistics test” (test and class anxiety), “trying to decide which analysis is appropriate for my research project” (interpretation anxiety), or “to ask a fellow student to explain a computer printout” (fear of asking for help). In an evaluation of its psychometric properties, Papousek et al. (2012) found a three-factor structure underlying a superordinate factor. This structure could be confirmed in a pre-study for the present research in which the psychometric properties of the translated version were analyzed in a sample of 1,061 psychology students (813 women, 245 men, 3 persons did not give information about their sex). Confirmatory factor analysis supported a three-factorial structure with a superordinate factor (*RMSEA* = 0.043, *CFI* = 0.908, *SRMR* = 0.054). These values can be considered as indicators of a good model fit (compare Papousek et al., 2012). The scales showed high consistency values in the present sample (Cronbach's α between 0.86 and 0.87).

Mathematics anxiety was measured by the Revised Mathematics Anxiety Ratings Scale (R-MARS; Baloglu and Zelhart, 2007). The R-MARS includes three subscales that measure mathematics test anxiety with 15, numerical task anxiety with 5, and mathematics course anxiety with 5 items. The self-report items require participants to describe on a five-point scale ranging from “no anxiety” (1) to “very much anxiety” (5) how they feel in specific situations, such as attending a mathematics examination. Examples for these situations are “taking an exam in a math course” (mathematics test anxiety), “being given a set of division problems to solve” (numerical task anxiety), or “listening to another student explain a math formula” (mathematics course anxiety). In a pre-study, the psychometric properties of the translated version were analyzed in a sample of 527 psychology students (380 women, 143 men, 4 persons did not give information about their sex). Confirmatory factor analysis supported a three-factorial structure with the scales described above (*RMSEA* = 0.05, *CFI* = 0.962, *SRMR* = 0.05). In the present sample, Cronbach's α ranged between 0.84 and 0.92.

In order to assess the relationship of the STARS and the R-MARS it was investigated beforehand by means of CFA whether the questionnaires belong to two separate latent factors or whether they can be subsumed under one latent factor. Results favor the solution with two separate latent factors and speak for the use of both instruments as originally intended by the developers and other researchers (Cruise et al., 1985; Baloglu and Zelhart, 2007; Papousek et al., 2012).

The general proneness to experience anxiety was measured by the State-Trait-Anxiety Inventory (STAI, German version; Laux et al., 1981). The trait anxiety scale consists of 20 items. Participants indicate how they generally feel and describe how often they experience anxiety-related feelings and cognitions on a 4-point rating scale ranging from “almost never” (1) to “almost always” (4) (item example: “I have disturbing thoughts”). Higher scale values indicate higher levels of trait anxiety. The total score is formed by the mean of answers on all items. The scale shows high internal consistency in the study sample of *n* = 225 (Cronbach's α = 0.92).

Academic procrastination was measured by the Procrastination Assessment Scale–Students (PASS; O'Callaghan et al., 2009; Macher et al., 2012, 2013). Participants indicate the degree to which they procrastinate on academic tasks in their statistics course, such as studying for the exam or keeping up with assignments [rating from “never procrastinate” (1) to “always procrastinate” (5)] and the degree to which procrastination on a task is a problem for them [rating from “not at all a problem” (1) to “always a problem” (5)]. The total score was calculated as the mean of answers on the eight items. This scale shows moderate internal consistency in the study sample of *n* = 225 (Cronbach's α = 0.71).

Concerning mathematics grades, the participants were asked about their last grade in mathematics at school. Lower values indicate better performance [scale of grade in the participants' school system ranges from very good (1) to not sufficient–fail (5)].

Academic performance was measured with a written statistics exam, which was administered at the end of the course. The 10 examination questions required cognitive operations ranging from reproduction of knowledge, via application of knowledge (e.g., calculations), to analyzing and explaining information (Anderson et al., 2001). The answers to the questions of the final exam were scored dichotomously (correct/not correct) so that participants could achieve a maximum of 10 points.

### Procedure

A design with three points in time was chosen for the study. Participants completed a demographic questionnaire, questions on grades in mathematics, the R-MARS, and the STAI at the beginning of term in October. Mathematics anxiety was measured at the start of term because various studies regard it as an antecedent of statistics anxiety (Baloglu, 2003). One week prior to the examination, at the end of term in January, all participants completed the STARS and measurements of procrastination by the PASS. These variables were measured at the end of term because students could gather experiences with their statistics course and the instructor and because some questions refer to recent learning behaviors. In the last week of term, students took the exam and their examination scores were recorded.

## Results

Table 1 displays the descriptive statistics of the variables of interest in the whole sample and for the subsamples of female and male students. Differences between female and male students were tested for all variables. Welch-tests show significant differences with regard to statistics test and class anxiety [*W*_(1, 100.624)_ = 11.138, *p* = 0.001, *d* = 0.515] and interpretation anxiety [*W*_(1, 127.738)_ = 14.212, *p* < 0.001, *d* = 0.518] with females reporting higher anxiety values. No differences are found for fear of asking for help [*W*_(1, 102.976)_ = 0.169, *p* = 0.682, *d* = 0.063]. According to the statistical results, females also have higher scores on mathematics test anxiety [*W*_(1, 108.636)_ = 7.849, *p* = 0.006, *d* = 0.418] and numerical task anxiety [*W*_(1, 185.454)_ = 28.270, *p* < 0.001, *d* = 0.629], but do not differ from males with regard to course anxiety [*W*_(1, 139.395)_ = 0.931, *p* = 0.336, *d* = 0.128]. With regard to all other variables, no differences between female and male students are found [trait anxiety: *W*_(1, 116.018)_ = 0.025, *p* = 0.876, *d* = 0.023; procrastination: *W*_(1, 91.486)_ = 1.792, *p* = 0.184, *d* = 0.219; academic performance: *W*_(1, 123.516)_ = 1.509, *p* = 0.222, *d* = 0.172; mathematics grades: *U* = 4256.000, *p* = 0.090, *d* = 0.256].

**Table 1:** Descriptive statistics for the variables investigated in the present study (total, female, and male students).

	***M***	***SD***	***MD***	**Min**	**Max**	***N***
Test and class anxiety	2.57	0.76	2.56	1.00	4.38	224
Female	2.67	0.73	2.75	1.13	4.38	164
Male	2.29	0.77	2.25	1.00	3.75	60
Interpretation anxiety	1.72	0.54	1.64	1.00	3.64	224
Female	1.80	0.56	1.73	1.00	3.64	164
Male	1.52	0.46	1.45	1.00	2.64	60
Fear of asking for help	2.10	0.82	2.00	1.00	4.75	224
Female	2.11	0.82	2.00	1.00	4.75	164
Male	2.06	0.84	2.00	1.00	4.75	60
Mathematics test anxiety	2.92	0.78	2.90	1.10	4.60	225
Female	3.01	0.79	3.00	1.20	4.60	164
Male	2.69	0.78	2.60	1.10	4.50	61
Numerical task anxiety	1.74	0.83	1.60	1.00	4.75	225
Female	1.88	0.88	1.80	1.00	4.75	164
Male	1.38	0.50	1.20	1.00	3.40	61
Mathematics course anxiety	1.40	0.54	1.20	1.00	4.00	225
Female	1.42	0.57	1.25	1.00	4.00	164
Male	1.35	0.44	1.00	1.00	2.40	61
Trait anxiety	2.06	0.51	1.95	1.05	3.70	222
Female	2.05	0.52	1.95	1.05	3.70	161
Male	2.07	0.48	2.00	1.30	3.30	61
Procrastination	2.55	0.62	2.63	1.00	4.50	225
Female	2.52	0.59	2.56	1.00	4.50	164
Male	2.65	0.70	2.63	1.38	4.00	60
Performance	5.50	1.99	5.50	1.00	10.0	225
Female	5.41	2.06	5.50	1.00	10.0	164
Male	5.75	1.78	5.50	1.00	9.00	61
Grades in mathematics			2.00	1.00	5.00	224
Female			2.00	1.00	5.00	163
Male			2.00	1.00	5.00	61

*Annotation: Grades were measured on an ordinal scale; therefore, M and SD were not calculated*.

Mathematics grades at school range between 1 and 5: 88.4% of students have a grade between 1 and 3 (22.2%: 1, 38.7%: 2); 9.8% just have passed their course with grade 4 and 1.8% have a fail (however, it is possible to receive a university entrance certificate with a fail).

Correlations between the investigated variables are presented in Table 2.

**Table 2:** Bivariate correlations between the variables investigated in the present study.

	**(2)**	**(3)**	**(4)**	**(5)**	**(6)**	**(7)**	**(8)**	**(9)**	**(10)**	**(11)**
Gender (1)	−0.223^**^	−0.224^**^	−0.028	−0.183^**^	−0.271^**^	−0.057	0.010	0.010	0.077	0.110
Test and class anxiety (2)	1.000	0.688^**^	0.489^**^	0.610^**^	0.278^**^	0.432^**^	0.357^**^	0.211^**^	−0.119	0.115
Interpretation anxiety (3)		1.000	0.515^**^	0.389^**^	0.375^**^	0.439^**^	0.281^**^	0.249^**^	−0.126	0.047
Fear of asking for help (4)			1.000	0.307^**^	0.231^**^	0.355^**^	0.275^**^	0.194^**^	−0.078	−0.002
Mathematics test anxiety (5)				1.000	0.367^**^	0.464^**^	0.327^**^	0.175^**^	−0.123	0.330^**^
Numerical task anxiety (6)					1.000	0.322^**^	0.124	0.106	−0.012	0.126
Mathematics course anxiety (7)						1.000	0.182^**^	0.146^*^	0.005	0.207^**^
Trait anxiety (8)							1.000	0.228^**^	0.050	0.038
Procrastination (9)								1.000	−0.247^**^	0.218^**^
Performance (10)									1.000	−0.290^**^
Grades in mathematics (11)										1.000

Significant correlations are marked with asterisks (

* *p < 0.05*,

** *p < 0.01)*.

**Figure 4:**
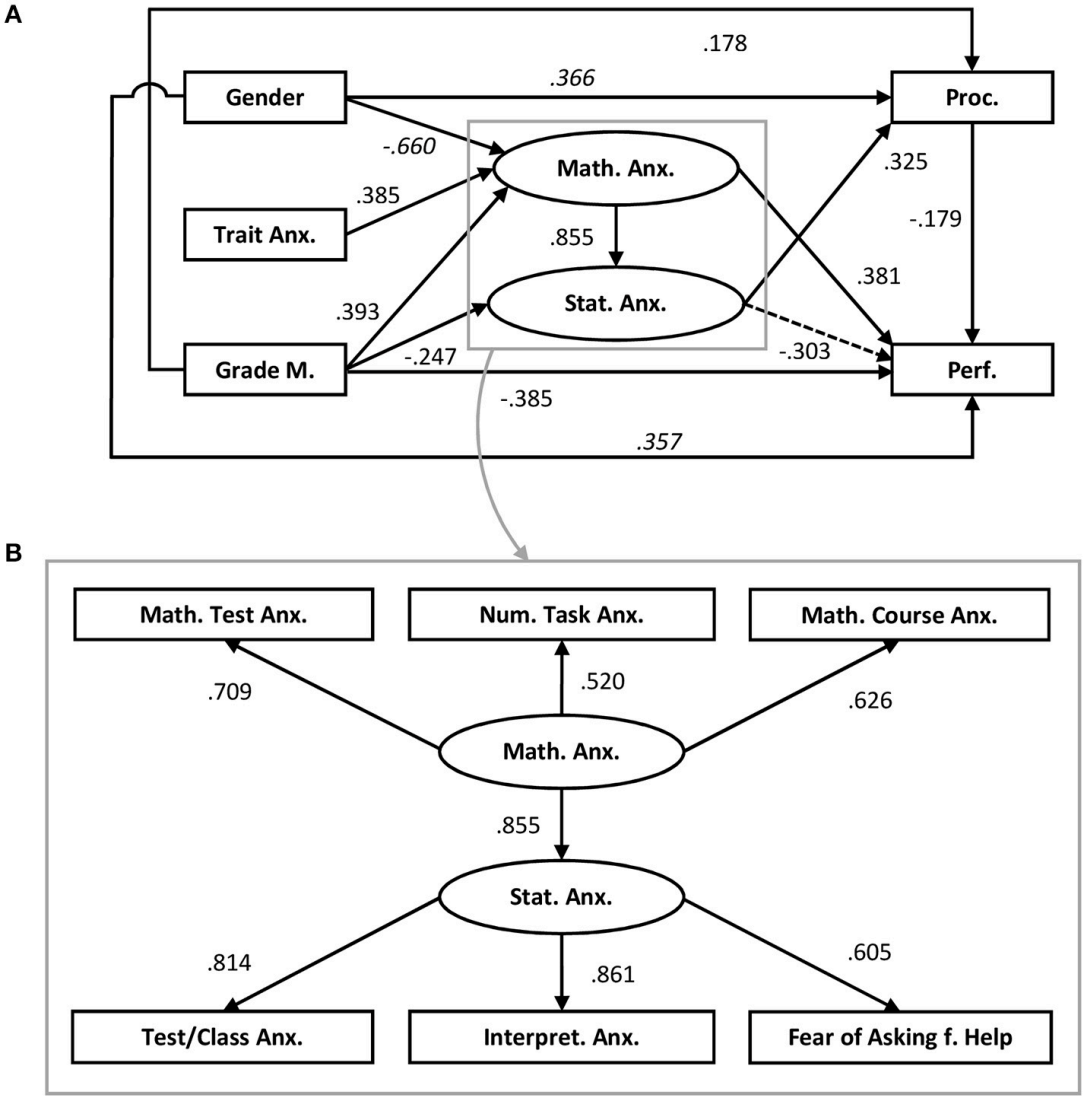
Result of Model 1 **(A)** Trait Anx., Trait anxiety; Grade M., Grades in mathematics; Math. Anx., Mathematics anxiety; Stat. Anx., Statistics anxiety; Proc., Procrastination; Perf., Performance in the statistics exam; **(B)** Math. Test Anx., Mathematics test anxiety; Num, Task Anx., Numerical task anxiety; Math. Course Anx., Mathematics course anxiety; Test/Class Anx., Test and class anxiety; Intepret. Anx., Intepretion anxiety; Fear of Asking f. Help, Fear of asking for help.

Three models which explain the relationship between mathematics anxiety and statistics anxiety and their role for performance were tested by means of structural equation modeling. It allows to examine direct as well as indirect effects of antecedents of mathematics anxiety and statistics anxiety on possible consequences and to test relationships between the variables in a multivariate context (compare Figure 4). The data were analyzed with Mplus 7 using a maximum likelihood estimator. The fit of the data to the hypothesized model and the quality of the model were assessed using χ^2^/df, CFI, SRMR, RMSEA, and adjusted BIC.

Results of Model 1 show a high β-weight for the path from mathematics anxiety to statistics anxiety. However, only a β-weight with a value of 1 would indicate that mathematics anxiety and statistics anxiety form one latent factor. This result already speaks against the alternative models 2 and 3. However, as the β-weight achieved a rather high value of 0.855 the two alternative models were calculated anyway. Fit indices and sample-size adjusted BIC for these models also suggest that Model 1 fits the data better than Models 2 and 3 (see Table 3, see Figure 4). Values of χ^2^/df < 2, CFI > 0.95, SRMR < 0.08, and RMSEA < 0.06 are considered indicators of good model fit (Hu and Bentler, 1999; Beauducel and Wittmann, 2005).

**Table 3:** Fit indices for the three structural equation models.

**Model**	**χ^2^**	**df**	**χ^2^/df**	**CFI**	**RMSEA**	**SRMR**	**Adj. BIC**
1	56.438	32	1.764	0.959	0.059	0.042	3657.273
2	118.416	34	3.483	0.859	0.106	0.053	3714.793
3	97.498	36	2.708	0.891	0.096	0.051	3698.333

In the following section, the results of Model 1 will be explained in more detail. Figure 4 shows the results of the structural equation model. In the structural equation model, two latent factors were formed, one for statistics anxiety, one for mathematics anxiety.

As Figure 4A shows, three variables are directly related to mathematics anxiety: Female students report higher levels of mathematics anxiety (β = −0.660). Participants with a higher propensity to experience anxiety in general report higher levels of mathematics anxiety (β = 0.385). Better grades in mathematics at school are related to lower mathematics anxiety (β = 0.393; lower values in grades indicate better performance). Altogether, 37.4% of the variance of the latent factor could be explained (*p* < 0.001).

Two variables are related to statistics anxiety: Grades in mathematics show a relation to statistics anxiety: better grades are related to higher statistics anxiety (β = −0.247). Mathematics anxiety is positively related to statistics anxiety (β = 0.855). Altogether, 63.4% of the variance of the latent factor of statistics anxiety can be explained (*p* < 0.001). Neither gender nor trait anxiety were related to statistics anxiety. Figure 4B belongs to the structural equation model and depicts the relationships between the components of mathematics anxiety and statistics anxiety. All facets of mathematic anxiety are significantly and strongly related to the latent factor; the same applies to statistics anxiety. Bivariate correlations between all facets are significant (see Table 2).

Three variables in the model are directly related to procrastination: Men are more prone to procrastination (β = 0.366). Poorer grades in mathematics at school are related to higher levels of procrastination (β = 0.178). Statistics anxiety is positively related to procrastination (β = 0.325). Altogether, 15.3% of the variance of the procrastination scores can be explained (*p* = 0.001).

Performance in the end-of-term statistics examination is related to five variables: Higher levels of procrastination (β = −0.179) and poorer grades in mathematics at school (β = −0.385) are related to poorer performance. Men perform better in the examination than women (β = 0.357). However, this relation between gender and performance is outweighed by men's higher tendencies to procrastinate, so that the mean performance values between genders do not differ from each other [*W*_(1, 123.516)_ = 1.509, *p* = 0.222, *d* = 0.172]. For statistics anxiety, only a marginally significant association is found: statistics anxiety is negatively related to performance (β = −0.303, *p* = 0.063). Higher levels of mathematics anxiety are related to better performance on the statistics examination (β = 0.381). Altogether, 17.3% of the variance of the performance values can be explained (*p* = 0.002).

## Discussion

### Overlap and differences between statistics anxiety and mathematics anxiety

Three structural equation models that differed with regard to the closeness of the relationship between mathematics anxiety and statistics anxiety were investigated. Altogether, the results of the models and their comparison speak for mathematics anxiety and statistics anxiety as two separate concepts with sub-facets. Model 1 which regards mathematics anxiety as an antecedent (and predictor) of statistics anxiety suggests that both forms of anxiety are highly correlated but not identical. Mathematics anxiety contributes with a high and significant weight of ß = 0.885 to statistics anxiety. Fit indices speak for Model 1.

Model 1 and the bivariate correlations between the sub facets (see Figure 4, Table 1) show that the sub-facets are related to each other, especially those sub-facets that measure anxiety in the same situation. Accordingly, the correlation between the test anxiety facets is highest with *r* = 0.610 (37.21% shared variance); the task-related facets correlate lower but still significantly with *r* = 0.389 (15.13% shared variance).

However, even though both types of anxiety share a large proportion of variance they also have genuine and even antagonistic contributions to academic performance. Altogether, the results of the study speak for two latent factors with shared but also unique components of statistics anxiety and mathematics anxiety.

### Antecedents of mathematics anxiety and statistics anxiety

The structural equation model shows three antecedents of mathematics anxiety: Female gender, a high proneness to experience anxiety in general, and poor grades in mathematics. Of the three antecedents, female gender is most strongly related to mathematics anxiety, a result that is in line with other studies (Meece et al., 1990; Devine et al., 2012; Macher et al., 2012, 2013; Cipora et al., 2015).

As expected, better grades in mathematics at school are related to lower levels of mathematics anxiety. This relationship may be explained by more positive experiences in mathematics-related situations that support confidence in the respective domain and counteract the development of mathematics anxiety.

The general proneness to experience anxiety to some extent also predisposes individuals to situation-specific anxieties. In the study, the general proneness to anxiety was measured as an antecedent of mathematics and statistics anxiety. It was chosen because statistics anxiety as well as mathematics anxiety describe content-related anxiety experienced in different situations. The results show that proneness to anxiety explains part of the variance of mathematics anxiety, but it has no incremental effect on statistics anxiety.

Concerning statistics anxiety, the structural equation model identifies two direct antecedents: good grades in mathematics and high levels of mathematics anxiety. Somewhat counterintuitively, grades in mathematics at school are directly and negatively related with statistics anxiety, meaning that students with better grades at school report higher levels of statistics anxiety. A possible explanation for this relationship might be that students realize over the course of their first term that mere knowledge and skills in higher secondary education mathematics are not sufficient to be successful in statistics. Statistics tasks are strongly embedded in an applied context and solving statistics tasks relies much more on verbal reasoning and drawing conclusions from data (Buck, 1987; Birenbaum and Eylath, 1994; Baloglu, 1999, 2003).

Therefore, it might be that as students with good grades in mathematics realize that this knowledge is only partially helpful in the statistics course they build up more anxiety. Grades, however, have antagonistic effects on statistics anxiety: a direct influence, by which better grades are related to higher statistics anxiety, plus an indirect effect via mathematics anxiety by which worse grades are related to higher mathematics anxiety levels and higher mathematics anxiety to higher statistics anxiety. The strongest predictor for statistics anxiety, however, is mathematics anxiety with a weight of β = 0.855.

### Contributions to academic performance

The bivariate correlations between the facets of mathematics anxiety and academic performance in the statistics examination are not significant. In the structural equation model, mathematics anxiety has a 2-fold, antagonistic impact on academic performance: a positive direct impact and a negative indirect impact via statistics anxiety on performance and via statistics anxiety and procrastination on performance. The positive impact of mathematics anxiety can probably be explained by students' motivational goals in the specific educational setting: According to Pekrun (1988), test anxiety—and therefore content-specific forms of anxiety as well—reduces positive intrinsic effort motivation. However, test anxiety may also induce motivation to avoid failure and the negative consequences of failure. Such failure-avoidance motivation may have different impacts on effort motivation. In situations where effort avoidance lacks negative consequences (e.g., in laboratory settings), individuals may avoid failure by not exhibiting any achievement behavior and thus prevent the risk to fail. In an educational setting, however, such effort avoiding behavior could pose a high risk. If students do not invest a minimum of effort and time preparing for the test, they will face severe consequences, such as failing the examination or even the whole degree. In that case, part of mathematics anxiety could have strengthened positive extrinsic effort motivation via the intention to avoid failure.

Studies by Wang et al. (2015) point to intrinsic motivation as a mediator for the relationship between mathematics anxiety and academic performance. When students are intrinsically motivated in mathematics moderate levels of mathematics anxiety are beneficial for achievement. Challenges in mathematics seem to induce these students to invest more effort into learning. In contrast, when intrinsic motivation in mathematics is low, a linear function between mathematics anxiety and achievement can be observed (Wang et al., 2015). These results suggest to look closer into motivational processes related to statistics and mathematics anxiety. In the present study, statistics anxiety has an indirect negative effect (via procrastination) and a direct negative effect on academic performance. Individuals with increased anxiety may experience low levels of self-efficacy and high fear of failure in potentially threatening situations (Haycock et al., 1998; Wolters, 2003; Rodarte-Luna and Sherry, 2008). Consequently, they tend to avoid certain tasks and situations, such as examinations, they postpone taking assignments or preparing for an examination and achieve less (Onwuegbuzie, 2004; Macher et al., 2012, 2013; Papousek et al., 2012). Furthermore, statistics anxiety has a direct negative influence on academic performance which, however, with *p* = 0.063 shows only a statistical trend. An explanation for this effect may be high levels of distress experienced during the examination. Macher et al. (2012) showed that statistics anxiety accounted for the maintenance of high distress levels throughout a written statistics examination.

It seems worth to carry out further investigations on the relationship between mathematics anxiety and performance in statistics. Research results on this relationship are ambiguous. In the presents study mathematics anxiety had a 2-fold and antagonistic effect on performance. In a study by Zeidner (1991) only statistics anxiety and not mathematics anxiety correlated with grades in statistics. In contrast, Pletzer et al. (2010) found a significant negative correlation between the two variables—however, only for students who responded with high increases in cortisol levels (as stress indicators) in the examination. These results point at variables that mediate the relationship between mathematics anxiety and performance in statistics. In the present study, statistics anxiety worked as mediating variable for the negative part of the relationship. Future studies should combine these different approaches to measure anxiety and stress and integrate physiological measures with self-reports as well as achievement variables. Also, further potential mediator variables should be investigated.

With regard to gender, the results confirm former studies in which women report higher levels of statistics anxiety (Rodarte-Luna and Sherry, 2008; Macher et al., 2012, 2013) and higher mathematics anxiety (Bieg et al., 2015; Cipora et al., 2015; Erturan and Jansen, 2015; Dowker et al., 2016). However, gender is related to a variety of variables in the model with different effects; for example, women seem to outweigh higher anxiety levels by favorable learning behaviors, such as low procrastination (Macher et al., 2012, 2013). Thus, in the present study, gender is not related to performance.

The two strongest predictors for good academic performance in statistics, however, are good former grades in mathematics with a bivariate correlation of *r* = −0.290 and a shared variance of 8.41% between both concepts and procrastination with a bivariate correlation of *r* = −0.247 and a shared variance of 6.10%. The former result supports research results by Fonteyne et al. (2015) in which results of a mathematics test contributed to passing a statistics course. Grades also had an indirect contribution to performance via procrastination. Students with better grades, and probably more positive learning experiences as well as more efficient learning behaviors, are less prone to procrastination.

### Implications for instruction

The results of the reported study do not only explain more of how anxiety is related to performance but are also important for instruction. They emphasize that a part of the achievements in statistics can be explained by prior mathematics success at school. One may ask which knowledge of mathematics at school is needed for success in the statistics examination. In a study on this question, Chiesi and Primi (2010) developed a test for basic mathematics abilities that they regard as essential for understanding statistics (with topics, such as fractions, equations, relations among decimal, and absolute numbers, probability rules etc.). In a sample of psychology students, students' results on the mathematics test but also statistics anxiety were related to success in the mid-term statistics exam. Fonteyne et al. (2015) developed a basic mathematics test for students of psychology and sociology measuring basic skills as for example numerical knowledge, operations with decimal numbers and brackets, fractions, percentages/proportions etc. Achievement in the basic mathematics tests correlated positively with the students' academic achievement including statistics. Various studies on secondary school students' knowledge and skills point out that students largely differ with regard to these basic skills (see for example PISA studies, Field, 2014; Organisation for Economic Co-operation Development, 2016) and this has consequences not only for later success in statistics but also for the development of positive attitudes and the prevention of anxiety in this domain. However, it should also be kept in mind that in the present study grades in mathematics and performance in statistics only shared 8.41% of variance. This small amount of variance can be explained by the different types of tasks and different cognitive processes that are needed in statistics, not only analytical tasks and processes but also probabilistic ones.

A similar amount of variance (6.10%) is shared between performance in statistics and procrastination. In that sense, the results emphasize the role of learning behaviors and the harmful impact of procrastination. They point out the importance of avoiding procrastination, of learning continuously throughout a course, of monitoring achievement continuously, and of filling gaps if necessary. Based on the results, instructors are advised to support anxious students already prior to the examination, for instance by regularly providing opportunities for exercise and reflection of learning content, by setting up several smaller tests and assignments instead of only one extensive test or assignment, and by fostering students' time management skills (Tuckman, 1998; Macher et al., 2013).

The results also emphasize that anxiety may have antagonistic effects on academic performance. On the one hand, it may impair performance while on the other hand it may be beneficial. This does not mean that instructors should instill anxiety into their students, but they also should not downplay the difficulty of the course and should emphasize the importance to put effort in it to finally succeed. Instructors may also emphasize the importance and the worth of statistics and describe clearly to students what they have to do in order to succeed in a course. If students understand the importance of an examination for their studies, and if they perceive that passing a course is possible and dependent on their skills and effort, they should be more motivated to invest effort in the exam preparation.

## Limitations of the study, directions for future research

When interpreting the results of the study, one has to consider that the investigated variables explain a significant, yet moderate amount of the variance in performance (17.3%). It is likely that the type of the examination influences how strongly students experience anxiety in the examination situation. In a comparison of written vs. oral examinations, Diaz et al. (2000) found stronger effects of anxiety in oral examinations. It would be worthwhile to conduct future studies to investigate the differential impact of the type of examination. In the present study, however, it was only possible to investigate written examinations because the curriculum of the investigated psychology students includes only written examinations.

## Author contributions

All authors listed have made a substantial, direct and intellectual contribution to the work, and approved it for publication

### Conflict of interest statement

The authors declare that the research was conducted in the absence of any commercial or financial relationships that could be construed as a potential conflict of interest.
